# Assessing the impact of privacy-preserving record linkage on record overlap and patient demographic and clinical characteristics in PCORnet^®^, the National Patient-Centered Clinical Research Network

**DOI:** 10.1093/jamia/ocac229

**Published:** 2022-11-30

**Authors:** Keith Marsolo, Daniel Kiernan, Sengwee Toh, Jasmin Phua, Darcy Louzao, Kevin Haynes, Mark Weiner, Francisco Angulo, Charles Bailey, Jiang Bian, Daniel Fort, Shaun Grannis, Ashok Kumar Krishnamurthy, Vinit Nair, Pedro Rivera, Jonathan Silverstein, Maryan Zirkle, Thomas Carton

**Affiliations:** Department of Population Health Sciences, Duke University School of Medicine, Durham, North Carolina, USA; Duke Clinical Research Institute, Duke University School of Medicine, Durham, North Carolina, USA; Department of Population Medicine, Harvard Medical School and Harvard Pilgrim Health Care Institute, Boston, Massachusetts, USA; Department of Population Medicine, Harvard Medical School and Harvard Pilgrim Health Care Institute, Boston, Massachusetts, USA; Datavant, San Francisco, California, USA; Duke Clinical Research Institute, Duke University School of Medicine, Durham, North Carolina, USA; Scientific Affairs, HealthCore, Inc., Wilmington, Delaware, USA; Department of Medicine, Weill Cornell Medicine, New York, New York, USA; Department of Medicine, Cook County Health and Hospital System, Chicago, Illinois, USA; Department of Pediatrics, Applied Clinical Research Center, Children's Hospital of Philadelphia, Philadelphia, Pennsylvania, USA; Department of Health Outcomes and Bioinformatics, College of Medicine, University of Florida, Gainesville, Florida, USA; Center for Outcomes and Health Services Research, Ochsner Health, New Orleans, Louisiana, USA; Regenstrief Institute, Indiana University, Indianapolis, Indiana, USA; Renaissance Computing Institute, University of North Carolina, Chapel Hill, North Carolina, USA; Humana, Sharon, Massachusetts, USA; OCHIN, Inc., Portland, Oregon, USA; Department of Biomedical Informatics, University of Pittsburgh, Pittsburgh, Pennsylvania, USA; Cohen Veterans Bioscience, New York, New York, USA; Louisiana Public Health Institute, New Orleans, Louisiana, USA

**Keywords:** privacy-preserving record linkage, distributed research networks, real-world data

## Abstract

**Objective:**

This article describes the implementation of a privacy-preserving record linkage (PPRL) solution across PCORnet^®^, the National Patient-Centered Clinical Research Network.

**Material and Methods:**

Using a PPRL solution from Datavant, we quantified the degree of patient overlap across the network and report a de-duplicated analysis of the demographic and clinical characteristics of the PCORnet population.

**Results:**

There were ∼170M patient records across the responding Network Partners, with ∼138M (81%) of those corresponding to a unique patient. 82.1% of patients were found in a single partner and 14.7% were in 2. The percentage overlap between Partners ranged between 0% and 80% with a median of 0%. Linking patients’ electronic health records with claims increased disease prevalence in every clinical characteristic, ranging between 63% and 173%.

**Discussion:**

The overlap between Partners was variable and depended on timeframe. However, patient data linkage changed the prevalence profile of the PCORnet patient population.

**Conclusions:**

This project was one of the largest linkage efforts of its kind and demonstrates the potential value of record linkage. Linkage between Partners may be most useful in cases where there is geographic proximity between Partners, an expectation that potential linkage Partners will be able to fill gaps in data, or a longer study timeframe.

## OBJECTIVE

The National Patient-Centered Clinical Research Network (PCORnet^®^) is a network-of-networks primarily funded by the Patient-Centered Outcomes Research Institute (PCORI) that utilizes standardized electronic health record (EHR) and administrative claims data to support multi-center observational and comparative effectiveness research.^1^^,^^2^ At the time of this work (September 2018–June 2021), PCORnet was comprised of 9 Clinical Research Networks (CRNs),^3–11^ 2 Health Plan Research Networks (HPRNs), and a Coordinating Center.^12^ CRN members include academic health centers, health systems, and federally-qualified health centers, which utilize EHRs as well as state/regional health plans with a payer relationship to one or more CRN clinical member sites, who provide administrative claims (“claims”). HPRNs are health plans with national coverage, each typically covering tens of millions of members. PCORnet functions as a distributed research network (DRN),^13^^,^^14^ in which Network Partners (network participants) harmonize their EHR and claims data contained within their source system(s) to the specifications of the PCORnet Common Data Model (CDM),^15^ with quarterly refreshes followed by a data curation process.^16^ Queries are distributed by the PCORnet Coordinating Center and the results are returned by Network Partners, typically as aggregate counts or summary statistics.

As stand-alone data sources, both EHRs and administrative claims suffer from certain limitations. EHRs can provide rich clinical detail on the care provided to patients within a health system, but may lack information on events that happen outside of a that health system.^17^^,^^18^ (In the United States, a health system can retrieve EHRs from outside health systems for patients for whom they provide care, but these external records cannot be used for research by the requesting health system.^19^) Administrative insurance claims contain complete outcomes for all medically-attended (and billed) events that occur related to that patient within an enrollment period regardless of where they received care, but they lack the level of granularity of an EHR.^20^ One way to address these gaps is through record linkage, bringing together a patient’s data from disparate sources to provide a more complete picture of their care.^21^^,^^22^ There are several different strategies for record linkage,^21–32^ but many healthcare data holders are gravitating toward privacy-preserving approaches, which support linkage without sharing direct patient identifiers.^22^^,^^23^^,^^25^^,^^28^

This article describes the implementation of the PCORnet privacy-preserving record linkage (PPRL) solution and the results from a demonstration study—to outline the degree of patient overlap across the network (how many patients had records with 1, 2, 3, or 4 or more Network Partners), and to create a de-duplicated summary that would describe the demographics and basic clinical characteristics of the PCORnet population with a clinical encounter in a participating health system.

## BACKGROUND AND SIGNIFICANCE

Several CRNs, particularly those with Network Partners concentrated in a single geographic area, had implemented record linkage solutions to allow for shared cohort identification and the creation of consolidated study records.^33^^,^^34^ As PCORnet conducted more cross-network activities that linked CRNs and HPRNs,^35^^,^^36^ it became clear that the use of multiple record linkage solutions was not sustainable. Each time an HPRN was asked to use a new record linkage tool, the proposed solution would need to go through an Information Technology (IT) security review, a contract review for software license agreements, a data governance review, etc., which often required months of discussion and negotiation. Adding in the ongoing personnel support costs made it difficult to execute linkage activities at scale. These factors were behind the efforts to adopt a common PPRL solution for PCORnet. The PCORnet PPRL solution was intended to help address 3 key scenarios: (1) de-duplication of patient data between CRN clinical sites due to care fragmentation, (2) data enrichment between CRNs and HPRNs, and (3) shared cohort discovery and analysis between CRNs and other sources (eg, patient registries).

PCORnet operates as a DRN, and its approach toward record linkage reflects this structure. The intent was not to develop a centralized linked repository of health data from all PCORnet patients for unspecified future research, but rather to establish the regulatory and technical infrastructure that would allow linkage to occur quickly and efficiently for specific projects. While the Coronavirus Disease 2019 (COVID-19) pandemic has since demonstrated that academic medical centers will agree to centralize and link data for pre-defined cohorts during a public health emergency,^37^ it is unlikely that they will agree to such a model for to-be-determined research projects that could involve their entire patient population.

PCORnet’s analysis represents one of the largest linkage efforts among academic medical centers, health plans, and other health systems. The initial analysis was primarily focused on understanding the degree to which patient records overlap between Network Partners as well as creating a de-duplicated table that summarizes the demographic and clinical characteristics of patients with a clinical encounter in a participating health system. In creating this summary, we also sought to understand the information gain that occurs in adding in data from linked administrative claims records to what is available within the EHR, as the ability to fill in gaps in data is one of the key value propositions behind record linkage.

## METHODS AND DATA AVAILABILITY

Throughout the rest of the article, we refer to DataMarts, which are the harmonized CDM resources that PCORnet Network Partners use to respond to queries. DataMarts are classified as EHR or claims-based depending on the underlying source system. HPRNs contain claims-based DataMarts, and CRNs are primarily EHR-based, though there are a handful of claims-based DataMarts within CRNs. Additional details about the methods and the results of an initial beta query can be found here.^38^

### Data availability

A dataset was created for this work, but the governance surrounding the project required that the patient-level data be destroyed after analysis. The underlying queries and source data exist to allow it to be recreated.

### Regulatory

A single common research protocol was written to govern the study activities and to provide consistent documentation for the Institutional Review Board (IRB) determination process. Across Network Partners, roughly half of their corresponding IRBs found the study to be non-human subjects research (*n* = 30), while slightly fewer viewed the study as exempt or covered under an existing IRB protocol (*n* = 27). Three (*n* = 3) Network Partners went through expedited IRB review, while only 1 (*n* = 1) had to submit the work for full IRB review.

### Tokens

PCORnet used a PPRL solution from Datavant.^39^ PCORnet Partners used the Datavant software to transform combinations and derivations of personally identifiable information (PII) extracted from their source systems into de-identified encrypted hash (ie, non-reversible) tokens. PII elements were selected based on their overall availability among PCORnet Partners,^40^ the utility of using tokens derived from these elements to identify overlap between patients, and a certification that the tokens were considered de-identified data elements under the Health Insurance Portability and Accountability Act (HIPAA) Privacy Rule using the Expert Determination method.^41^ Partners were provided guidance on how to format the PII inputs (eg, remove titles [Mr., Mrs., Ms.] and middle initials from names) as well as the validation rules of the tokenization software. Partners used local staging tables to house the input PII for each patient as well as the tokenized output of the Datavant software. These tokens were then loaded into the HASH_TOKEN table of each DataMart’s PCORnet CDM.

### Queries

Two queries were distributed to participating DataMarts. The first query extracted the encrypted hash tokens for all patients in each DataMart’s PCORnet CDM (*full population cohort*). EHR DataMarts define the overall inclusion criteria for their CDM slightly differently (often based on when their contributing health system(s) implemented their EHR), but they generally include at least all patients with a clinical encounter at the health system(s) that contribute data to the EHR on or after January 1, 2009. The second query generated demographic and clinical characteristics for all patients with a clinical encounter between January 1, 2018 and December 31, 2019 (*2018–2019 cohort*). Variables included age group (10-year groupings calculated based on patient age at the time of query), gender (administrative sex), race, and binary indicators for clinical characteristics that indicated presence or absence, within the period of 2018–2019, of selected co-morbidities (ie, asthma, cancer, dementia, depression, type 2 diabetes, heart failure), a lipid-lowering medication (eg, order, dispensing or administration), and a result for a low-density lipoprotein (LDL) laboratory test. This smaller cohort was created because PCORnet Network Partners were not comfortable sending these patient-level variables for the full population cohort to the Coordinating Center for analysis.

Each query separately produced a patient-level dataset that is considered de-identified under HIPAA. An additional Expert Determination would be required to determine that the combined dataset was also de-identified. When using the Expert Determination standard on combined datasets, a statistical risk assessment is performed to ensure the aggregated data is statistically de-identified (ie, a qualified statistical expert has determined the risk of re-identification is very small).^41^ To avoid the time and cost of this assessment and to support efficient distributed analytics, we implemented workflows to keep the de-identified tokens separate from the clinical and demographic characteristics.^42^

**Figure 1.**
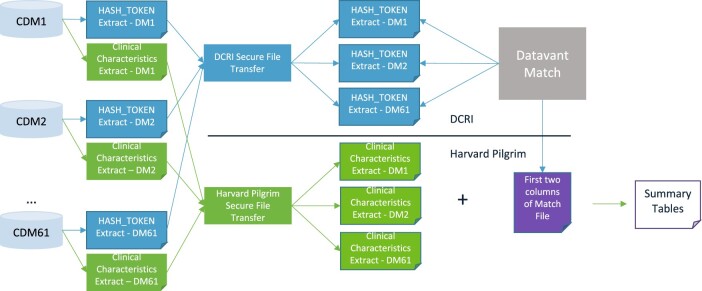
Flow of data from each PCORnet Data Mart to the Coordinating Center. The HASH_TOKEN extract goes to DCRI for processing and then is matched using the Datavant software. The match IDs are then sent to Harvard Pilgrim for linking with the clinical characteristics for further processing and analysis.

PCORnet Network Partners returned the token extract to the arm of the PCORnet Coordinating Center supported by the Duke Clinical Research Institute (DCRI), while the demographic and clinical characteristics were returned to the portion supported by the Harvard Pilgrim Healthcare Institute (HPHCI). This separation of roles is a linkage best practice that ensures that no individual party within the PCORnet Coordinating Center would work with the combined dataset. A visualization of this workflow is shown in Figure 1.

### Determining overlap and analysis

The DCRI PCORnet Coordinating Center team processed the hash tokens from each partner using the Datavant software and executed a Match algorithm to determine overlap, generating a match index (ie, de-identified master patient index) for the entire network. The DCRI team transferred a version of the match index without the hash tokens to the HPHCI team so that it could be linked to the demographic and clinical characteristic data and to complete the analyses described below.

We assessed the overlap of patients among PCORnet DataMarts, calculating the percentage of patients who appeared in 1, 2, 3, or ≥4 DataMarts as well as the percentage of matched patients within each DataMart to assess potential within-DataMart duplicates. The within-DataMart duplicates were removed and we then calculated the number and percentage of patients that overlapped for every pairwise combination of DataMarts. We completed these calculations for both the full population cohort and the 2018–2019 cohort. We also performed a sensitivity analysis to determine the impact on matching rates between one CRN and HPRN when using the full set of available tokens compared with a single token.

We generated a summary-level table of patient demographic and clinical characteristics for patients in the 2018–2019 cohort with a record in an EHR-based DataMart. We also looked at the potential information gain by adding overlapping records for the subset of EHR-based patients who also have data in a claims-based DataMart during the same 2018–2019 period. We applied a series of adjudication steps to consolidate matched records while retaining distinct information among records with discrepant values. A comorbidity, medication, or laboratory record present in any record at any DataMart within the observation period (2018–2019), was defined as “present” in the consolidated record (otherwise “not present”). If demographic variables were unique due to discrepant values (eg, gender of M (Male) in one record and F (Female) in a matched record), the final record value was defined as “discordant.” Otherwise, we used the known values from the linked DataMarts(s).

## RESULTS

### Overall query results

A total of 61 DataMarts responded to the overlap queries (59 CRN-based and 2 HPRN-based). A Match file was generated using the Datavant Match software. We declared 2 records to be a match if a majority of available tokens were the same (net_tokens). In other words, if 6 tokens were present, 4 needed to match, if 4 or 5 were present, 3 needed to match. The availability of tokens across the network is shown in Table 1 as the median percentage of the population with a non-error token across all contributing DataMarts. Also shown is the input PII for each token and the range for lowest and highest percentage availability across all DataMarts. We had originally planned to use logic that would declare 2 records to be a match if any of the SSN-based tokens were the same before defaulting to the majority-matching approach (net_tokens_ssn), but this resulted in significant performance degradation in the version of Datavant Match that we used at the time (v3.6), with processing speed falling to tens of records per second compared with tens of thousands. We believe that this is due to some Network Partners that generated tokens using dummy SSNs for a large number of patients, which caused a resulting duplication of the SSN-based tokens and the creation of artificially large clusters of matched patients.

**Table 1.** Median percentage of tokens populated across DataMarts and the personally identifiable information (PII) used to generate each one, along with the range of high and low values

Token (Input PII)	Median percentage range (low, high)
Token 1 (last name, 1st initial of first name, gender, date of birth)	100% (100, 100)
Token 2 (last name [soundex], first name [soundex], gender, date of birth)	100% (100, 100)
Token 3 (last name, first name, date of birth, 3-digit zip code of current address)	99% (77, 100)
Token 4 (last name, first name, gender, date of birth)	100% (100, 100)
Token 5 (social security number, date of birth, gender)	67% (0, 99)
Token 16 (social security number, first name)	66% (0, 99)

The numbers for the full population and 2018–2019 cohorts were computed after removing the within-DataMart overlaps (ie, internal duplicates). The percentage of internal duplicates within DataMarts was as follows: less than or equal to 1%, *n* = 43 DataMarts; between 1 and 5%, *n* = 14; greater than 5%, *n* = 2. Overall, within the full population cohort, there were ∼170M patient records across the 61 submitting DataMarts, with ∼138M (81%) of those corresponding to a unique patient based on the Datavant matching algorithm. Within the 2018–2019 cohort, there were 72.2M patient records in total across the 61 DataMarts, with 65.1M being unique (90.2%). In terms of overlap across DataMarts, within the full population cohort (∼138M unique), 82.1% of patients were found in a single DataMart and 14.7% were in 2 DataMarts. Within the 2018–2019 cohort, 90.5% of patients were found in a single DataMart and 8.5% were in 2 DataMarts. This information is summarized in Table 2.

**Table 2.** Overall number of patient records between cohorts (full CDM, visit in 2018–2019), as well as the number of unique patients

	Full CDM	Visit in 2018–2019
**Total number of patient records (all responding DataMarts)**	170 387 769	72 221 222
**Unique patients**	138 753 666	65 060 458
**Number of DataMarts in which a patient record is present**		
** 1**	82.1%	90.5%
** 2**	14.7%	8.5%
** 3**	2.6%	0.9%
** ≥4**	0.6%	0.1%

*Note*: Also shown is the percentage of patient records in 1, 2, 3, or more DataMarts for each cohort.

### DataMart-to-DataMart overlap

The percentage overlap between DataMarts in the 2018–2019 cohort varied greatly, with a range between 0% and 80% and a median of 0%. The total number of patient records that overlapped between DataMarts in this cohort ranged from 0 to 361K. To assess the most significant overlap, we examined the DataMart pairs where the overlap was greater than 10% of the population (using either site as denominator) or totaled more than 75K patients (*n* = 41). We found that these pairs fell into one of the following categories: geographical proximity between unaffiliated health systems (eg, healthcare facilities within 10–20 miles of one another; *n* = 18), health systems that provided care for a significant number of patients with insurance from an associated health plan (this includes CRN-HPRN linkage or within-CRN linkage for CRNs that included health systems and health plans; *n* = 21), or an underlying organizational relationship (ie, health system and affiliated clinic network; *n* = 2). The number of patients with overlapping records in the “geographic proximity” category was roughly the same as that of the “health system-health plan” category.

### Token sensitivity analysis

Two of the hash tokens used for linkage were partially based on SSN. In addition to the quality issues with SSN noted above, many CRNs were missing SSNs entirely, either because health systems stopped the practice of collecting SSNs,^40^ or the research team chose not to seek institutional approval to use available SSNs in the token generation process, which resulted in missing SSN-based tokens (*n* = 13 DataMarts in total). Some of the CRNs expected a higher overlap with the HPRNs and questioned whether these missing SSN-based tokens could influence the performance of the matching algorithm. To test this, we conducted a sensitivity analysis of the overlap between 1 CRN and 1 HPRN using all available tokens compared with a single token that was based on patient first and last name, gender and date of birth (Token 4). Tokens 1–4 were present in >99% of patient records. Within the HPRN, the SSN-based tokens (Tokens 5 and 16) were present in ∼89% of patient records. Within the CRN, the percentages for the SSN-based tokens were 25%, 48%, 55%, 62%, and 69% (ordered smallest to largest). The results of this analysis are shown in Table 3, with almost identical matching performance between all available tokens and the single token, differing by 0.1% at a handful of Network Partners within the CRN.

**Table 3.** Overlap between Network Partners of a CRN and one HPRN for the full CDM cohort for a single token [Token 4] (a), and all tokens [Tokens 1, 2, 3, 4, 5, 16] (b)

(a)						
% Patients in common—single token used in match						
DataMart	CRN site 1	CRN site 2	CRN site 3	CRN site 4	CRN site 5	HPRN
**CRN site 1**	100.0%	14.2%	20.7%	19.1%	29.9%	19.4%
**CRN site 2**	16.2%	100.0%	11.2%	6.8%	9.4%	12.4%
**CRN site 3**	16.4%	7.8%	100.0%	25.0%	21.5%	19.1%
**CRN site 4**	11.5%	3.6%	19.0%	100.0%	19.2%	22.3%
**CRN site 5**	23.2%	6.4%	21.0%	24.7%	100.0%	21.6%
**HPRN**	0.8%	0.5%	1.0%	1.6%	1.2%	100.0%

(b)						
% Patients in common—all tokens considered for match						
DataMart	CRN site 1	CRN site 2	CRN site 3	CRN site 4	CRN site 5	HPRN
**CRN site 1**	100.0%	14.1%	20.7%	19.1%	29.9%	19.3%
**CRN site 2**	16.1%	100.0%	11.2%	6.7%	9.4%	12.3%
**CRN site 3**	16.4%	7.7%	100.0%	25.0%	21.5%	19.0%
**CRN site 4**	11.5%	3.5%	19.0%	100.0%	19.2%	22.2%
**CRN site 5**	23.1%	6.3%	21.0%	24.6%	100.0%	21.5%
**HPRN**	0.8%	0.5%	1.0%	1.6%	1.2%	100.0%

**Figure 2.**
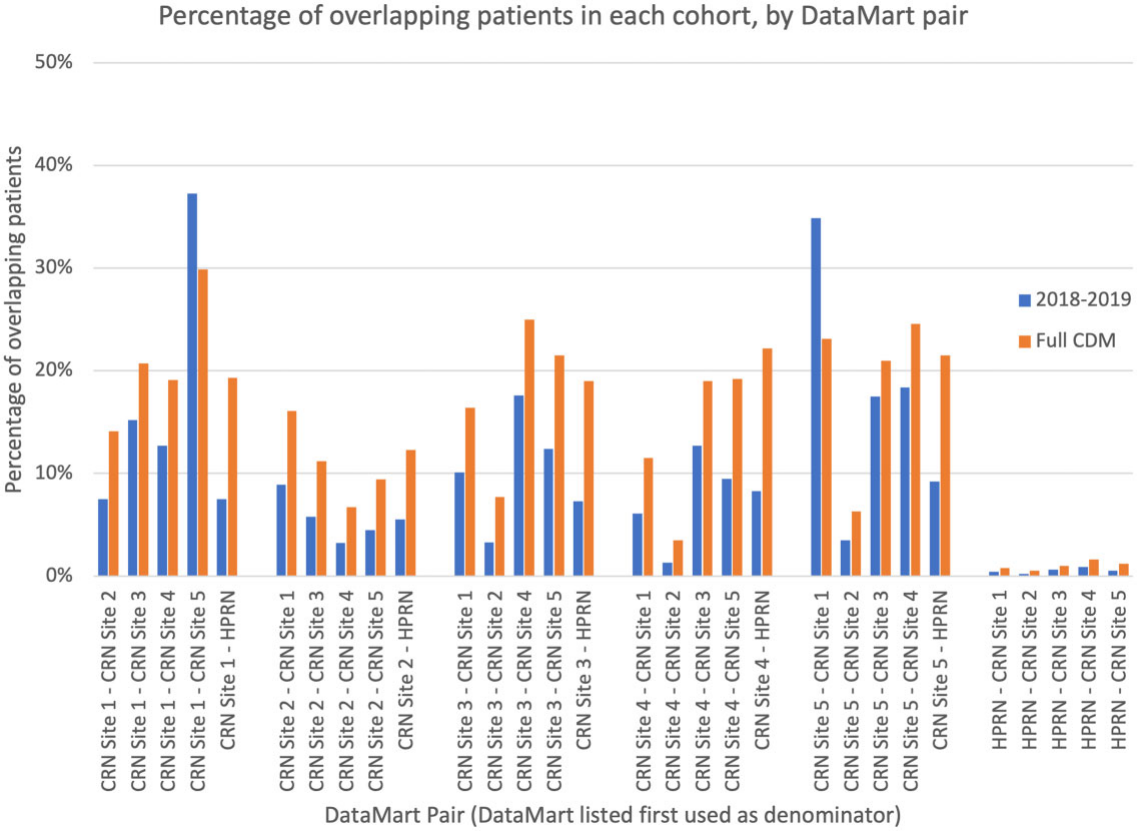
Overlap between each DataMart in a CRN and HPRN for different cohorts (full CDM and visit in 2018–2019).

The overlap is much lower when looking within a small timeframe (eg, 2018–2019) compared with all years of data represented within each DataMart (typically 10+ years). This is shown in Figure 2, which shows the overlap for every pair of DataMarts within the same CRN and HPRN. There is a lower overlap for the 2018–2019 cohort for all pairs but one between CRN Network Partners (this pair is geographically proximal and shares an organizational relationship). For the pairs involving the HPRN, this lower overlap is likely due to the fact that patients switch health plans relatively frequently (eg, every 3 years), making the likelihood of health plan overlap smaller for any specific year compared with all available years (even with a small overlap percentage, HPRNs may have records on tens of millions of patients, making the overall number of matched patients relatively large). Similar behavior was seen when looking at the broader network overlap discussed previously (81% of patients in a single DataMart in the overall cohort, compared with 90% of patients in the 2018–2019 cohort).

### Demographics and clinical characteristics

To obtain a better understanding of the basic demographics and clinical characteristics of the network, we created a de-duplicated cohort of patients with a visit in an EHR-based DataMart between 2018 and 2019 (participants in PCORnet studies tend to originate from the EHR-based DataMarts). This population, totaling ∼40M patients, is shown in Table 4. For the subset of patients within this EHR visit-based cohort who also have records in one of the claims-based DataMarts (*n* = ∼3.1M), we also looked at the potential information gain, in terms of relative and absolute difference, by incorporating those claims data into the summary table.

**Table 4.** Demographic and clinical characteristics for patients with a visit in 2018–2019 and a record in an EHR-only DataMart (EHR Only Records column), as well as those EHR patients who also have an available administrative claim (EHR Patients with Available Claims)

	EHR only records—all EHR DataMarts		EHR patients with available claims (unlinked)		EHR patients with available claims (linked)		Relative difference (EHR + claims linked) %	Prevalence absolute difference (EHR + claims linked) %
	*N*	%	*N*	%	*N*	%		
Number of patients	39 758 272	100.0%	3 136 916	100.00%	3 136 916	100.00%		
**By age (*N*, % of patients)**								
0–11	6 562 669	16.5%	688 540	21.95%	688 498	21.95%	−0.01%	0.00%
12–19	4 121 678	10.4%	390 875	12.46%	390 823	12.46%	−0.01%	0.00%
20–34	7 117 259	17.9%	490 265	15.63%	490 182	15.63%	−0.02%	0.00%
35–49	6 845 564	17.2%	434 154	13.84%	434 098	13.84%	−0.01%	0.00%
50–64	7 602 760	19.1%	497 682	15.87%	497 602	15.86%	−0.02%	0.00%
65–74	4 276 626	10.8%	366 512	11.68%	366 434	11.68%	−0.02%	0.00%
75+	3 230 924	8.1%	268 712	8.57%	268 700	8.57%	0.00%	0.00%
Discordant	792	0.0%	176	0.01%	579	0.02%	69.60%	0.01%
**By gender (*N*, % of patients)**								
Female	22 081 901	55.5%	1 788 085	57.00%	1 788 408	57.01%	0.02%	0.01%
Male	17 650 657	44.4%	1 347 978	42.97%	1 348 299	42.98%	0.02%	0.01%
Other/missing	25 176	0.1%	802	0.03%	0	0.00%	0.00%	−0.03%
Discordant	538	0.0%	51	0.00%	209	0.01%	75.60%	0.01%
**By Race (*N*, % of patients)**								
American Indian or Alaska Native	129 007	0.3%	5842	0.19%	5070	0.16%	−15.23%	−0.02%
Asian	1 298 799	3.3%	69 476	2.21%	72 708	2.32%	4.45%	0.10%
Black or African American	6 064 456	15.3%	541 284	17.26%	566 414	18.06%	4.44%	0.80%
Native Hawaiian or Other Pacific Islander	89 864	0.2%	3978	0.13%	3117	0.10%	−27.62%	−0.03%
White	24 330 629	61.2%	1 926 990	61.43%	1 996 518	63.65%	3.48%	2.22%
Other/missing	7 790 838	19.6%	581 325	18.53%	463 344	14.77%	−25.46%	−3.76%
Discordant	54 679	0.1%	8021	0.26%	29 745	0.95%	73.03%	0.69%
**Recorded History of:**								
Asthma	2 044 213	5.1%	180 025	5.74%	419 411	13.37%	132.97%	7.63%
Cancer	2 018 535	5.1%	158 717	5.06%	295 813	9.43%	86.38%	4.37%
Dementia	524 912	1.3%	47 998	1.53%	106 608	3.40%	122.11%	1.87%
Depression	2 506 481	6.3%	189 116	6.03%	516 725	16.47%	173.23%	10.44%
Type 2 diabetes	2 741 251	6.9%	236 894	7.55%	434 077	13.84%	83.24%	6.29%
Heart failure	858 864	2.2%	82 455	2.63%	177 138	5.65%	114.83%	3.02%
Low-density lipoprotein lab	7 581 514	19.1%	581 385	18.53%	947 712	30.21%	63.01%	11.68%
Lipid-lowering medication	3 949 284	9.9%	306 372	9.77%	572 451	18.25%	86.85%	8.48%

For these patients, we provide summary results for both the unlinked (EHR only) and linked cohorts (EHR + claims). We describe the information gain through linkage in terms of relative difference (difference between EHR and EHR + claims divided by EHR + claims value for each variable) and absolute difference (difference between EHR and EHR + claims divided by the overall cohort).

While there was essentially no change in the overall percentages for the cohort related to age and gender demographics, we saw a decrease in the number of patients with values of other/missing. We also observed a decrease in other/missing values for race, but an increase in discordant records that contributed to a shift in distribution, particularly for race categories with the lowest overall counts. With the clinical characteristics, we see an increase in every concept, with a ∼63% to ∼173% increase in relative difference, and an absolute difference increase between 1.9% and 11.7%. The smallest absolute increases occur in dementia and heart failure, which are also the concepts with the fewest patients. The largest relative increases are found in asthma, dementia, depression, and heart failure, while the largest absolute increases occur with depression and the presence of an LDL lab result.

## DISCUSSION

Our results demonstrate the potential benefit for enriching PCORnet CRN data through PPRL, as well as important caveats. There was a high degree of variability in the overlap between DataMarts, with the highest percentage occurring between those organizations that are geographically proximate or have some sort of organizational or health plan relationship. The overlap between geographically distinct health systems was low as a percentage of their overall populations (often 0%), but often contained a non-zero number of patients. The tokens used to match patients did not have much of an impact on the overlap percentage, but it did vary with by time (overlap increases as the length of time increases).

The overlap between DataMarts varied substantially between the overall and 2018–2019 cohorts, with a reduction of >50% in some cases. This is an important consideration when designing a linkage study. If linking CRNs and HPRNs to find outcomes within a 1–2 year window, there will be a smaller degree of overlap, so more health plan Partners may be necessary to cover a patient population. On the other hand, if the purpose of linkage is to obtain information on baseline clinical characteristics that were present in the 5 years prior to an index event, fewer linkages may be necessary, given that overlap increases with time.

We generated tokens on the most recent demographic data held by PCORnet Partners. We found the greatest success when linking with tokens based on first and last name, gender, date of birth, and current zip code (Tokens 1–4). SSN can be a very precise identifier to use in linking, but only if the data are relatively clean. As part of this project, we did not specifically ask Network Partners to remove dummy identifiers, which impacted processing performance (Datavant has updated their software to remove such identifiers before tokenization). Health systems are moving away from the collection of SSN, and given that the remaining tokens are all derived from variations of the same PII, we may also need to consider additional tokens based on other invariant identifiers (eg, cell phone number or e-mail address) or tokens that include historical demographic information to better link records over a longer time periods. Other network initiatives are beginning to explore the use of an expanded set of tokens,^43^ which can inform our work.

The summary table of demographics and clinical characteristics demonstrates the benefit of linking EHR and administrative claims. There was very little increase in discordance for demographic records, aside from race, which has known issues with data capture, particularly within administrative claims.^44^^,^^45^ After linkage, we saw increases in the prevalence across all clinical characteristics. Given that most of the characteristics in our summary table are describing somewhat chronic conditions, we would likely seen an even bigger increase if we examined acute events or conditions that might occur out of system, and therefore be missing from an EHR-based DataMart (eg, myocardial infarction, ischemic stroke). This is evident by the fact that we saw the largest absolute increases in prevalence with depression and the presence of a result from an LDL laboratory test. Mental health conditions are often treated outside of traditional health systems (eg, therapists, social workers), and diagnoses associated with those visits might be more likely to show up in an external claim within an HPRN. With laboratory tests, results from outside facilities may be shared for treatment and care coordination, but the receiving facility may not be able to use those data for its own research. Therefore, record linkage can be particularly useful for studies that need outcomes or baseline clinical characteristics that are more likely to fall into this “out of system” category (care received outside of the health system providing data for research). If full population linkage is not feasible, it is increasingly important to include at least a subset of patients with linked EHR and claims for a sensitivity analysis of clinical findings.

## CONCLUSIONS/LIMITATIONS/FUTURE WORK

We successfully demonstrated the ability to use PPRL to identify overlap across ∼170M patient records and created a de-duplicated summary of demographic and clinical characteristics for patients with a visit in 2018 or 2019 using data from 61 Network Partners. This project was one of the largest linkage efforts of its kind. The overlap between DataMarts was highly variable, so the decision on whether to use record linkage should be based on the necessary data elements, the population and time frame under consideration, and characteristics of the contributing Network Partners. Linkage between CRNs may be more useful in cases where there is geographic proximity between Partners, an expectation that potential linkage Partners will be able to fill gaps in data, or a longer study timeframe. We are now working to deploy Datavant as part of several rare disease studies being funded by PCORI that are linking records between health systems and health plans.

One limitation of this project was that we did not conduct a validation of the linkage algorithm itself by comparing the matched charts at different health systems to confirm they were the same patient. These analyses have occurred elsewhere,^33^^,^^46–49^ but were not a component of this work. In addition, while we were able to show that supplementing EHR data with administrative claims from health plans resulted in an information gain related to prevalent conditions, governance challenges limited the scope of data that could be used in our analysis (number of variables and timeframe). A more robust characterization of clinical features would illustrate the full breadth and depth of this linkage.
